# Multimodal in-vivo maps as a tool to characterize retinal structural biomarkers for progression in adult-onset Stargardt disease

**DOI:** 10.3389/fopht.2024.1384473

**Published:** 2024-04-23

**Authors:** Hilde R. Pedersen, Stuart J. Gilson, Lene A. Hagen, Josephine Prener Holtan, Ragnheidur Bragadottir, Rigmor C. Baraas

**Affiliations:** ^1^ National Centre for Optics, Vision and Eye Care, Faculty of Health and Social Sciences, University of South-Eastern Norway, Kongsberg, Norway; ^2^ Department of Ophthalmology, Oslo University Hospital, Oslo, Norway; ^3^ Faculty of Medicine, University of Oslo, Oslo, Norway

**Keywords:** Stargardt disease, STGD1, *ABCA4*, adult-onset, multi-modal imaging, adaptive optics, retina, photoreceptors

## Abstract

**Purpose:**

To characterize retinal structural biomarkers for progression in adult-onset Stargardt disease from multimodal retinal imaging in-vivo maps.

**Methods:**

Seven adult patients (29–69 years; 3 males) with genetically-confirmed and clinically diagnosed adult-onset Stargardt disease and age-matched healthy controls were imaged with confocal and non-confocal Adaptive Optics Scanning Light Ophthalmoscopy (AOSLO), optical coherence tomography (OCT), fundus infrared (FIR), short wavelength-autofluorescence (FAF) and color fundus photography (CFP). Images from each modality were scaled for differences in lateral magnification before montages of AOSLO images were aligned with en-face FIR, FAF and OCT scans to explore changes in retinal structure across imaging modalities. Photoreceptors, retinal pigment epithelium (RPE) cells, flecks, and other retinal alterations in macular regions were identified, delineated, and correlated across imaging modalities. Retinal layer-thicknesses were extracted from segmented OCT images in areas of normal appearance on clinical imaging and intact outer retinal structure on OCT. Eccentricity dependency in cell density was compared with retinal thickness and outer retinal layer thickness, evaluated across patients, and compared with data from healthy controls.

**Results:**

In patients with Stargardt disease, alterations in retinal structure were visible in different image modalities depending on layer location and structural properties. The patients had highly variable foveal structure, associated with equally variable visual acuity (-0.02 to 0.98 logMAR). Cone and rod photoreceptors, as well as RPE-like structures in some areas, could be quantified on non-confocal split-detection AOSLO images. RPE cells were also visible on dark field AOSLO images close to the foveal center. Hypo-reflective gaps of non-waveguiding cones (dark cones) were seen on confocal AOSLO in regions with clinically normal CFP, FIR, FAF and OCT appearance and an intact cone inner segment mosaic in three patients.

**Conclusion:**

Dark cones were identified as a possible first sign of retinal disease progression in adult-onset Stargardt disease as these are observed in retinal locations with otherwise normal appearance and outer retinal thickness. This corroborates a previous report where dark cones were proposed as a first sign of progression in childhood-onset Stargardt disease. This also supports the hypothesis that, in Stargardt disease, photoreceptor degeneration occurs before RPE cell death.

## 1 Introduction

Multimodal ophthalmic imaging, including adaptive optics scanning light ophthalmoscope (AOSLO) imaging, presents clinicians and researchers with quantifiable but complex retinal structural information. Semi-automatic methods for correlating data across modalities, while preserving the spatial relations between images, enables more accurate identification, delineation, and comparison of normal and abnormal changes in retinal structure, down to single cell level (1, 2). In inherited macular dystrophies, this has the potential to improve differential diagnosis thereby facilitating correct diagnosis. And as such, allowing for improved understanding of disease progression and correct identification and selection of patients for clinical treatment trials.

Early and correct diagnosis is of particular importance for Stargardt disease (STGD1), a common inherited macular dystrophy caused by mutations in the *ABCA4* gene, and especially for adult-onset STGD1 (age of onset ≥ 20 years; late-onset STGD1 is a subtype of adult-onset STGD1 if age of onset is 45 years or older). Late-onset STGD1 is believed to be underdiagnosed as it can be difficult to differentiate between STGD1 and other macular dystrophies with adult- or late onset (3). The most common misdiagnosis given is age-related macular degeneration (AMD) (4, 5) and it is assumed that some patients with STGD1 may be unintentionally enrolled in AMD clinical trials (3). The phenotypic variability in *ABCA4*-associated STGD1 is reported to be large (6, 7), and some structural and functional changes appear to be similar between STGD1 and AMD. Thus, retinal structural changes that are used to differentiate adult-onset STGD1 from AMD for example (such as the appearance of macular or peripheral yellow-white flecks present in the transition between retinal pigment epithelium (RPE) and photoreceptor outer segments), may not be the earliest biomarker and is not present in all patients at the first visit [(3), see Supplementary Table S3].

The pathological steps by which mutations in the *ABCA4* gene lead to clinically detectable RPE changes remain unclear. The *ABCA4* gene encodes a protein localizing to photoreceptor outer segments (8, 9) and RPE (10). In childhood-onset STGD1, parafoveal atrophy with foveal sparing is reported to be the first sign, sometimes predated by foveal intraretinal yellow-white dots (11). In two case studies on childhood-onset STGD1, AOSLO imaging revealed photoreceptor loss to precede any structural changes in the RPE (12, 13). Further to this, they reported that intact photoreceptor inner segments underlying non-reflecting dark cones may be an early biomarker of childhood-onset STGD1 (12). As of yet, there are few reports on dark cones in adult-onset STGD1 multimodal in-vivo AOSLO imaging. Our hypothesis was that dark cones may be an early biomarker of STGD1 disease progression, irrespective of onset.

Employing AOSLO and custom software, we acquired images and constructed multimodal in-vivo maps to assess retinal structural biomarkers for progression in adult-onset STGD1. Specifically, we combined objective measurements of eye biometry and retinal magnification factor calculations to transform all imaging modalities to a common scale. Then, via montaged AOSLO images, we spatially aligned the other imaging modalities such that there was a high-precision alignment between pixels across modalities. This allowed us to directly compare normal versus abnormal retinal structures, to identify dark cones and other retinal alterations and assess their location and correlation with structural changes in different retinal cell layers.

## 2 Materials and methods

### 2.1 Participants

The study was undertaken at the National Centre for Optics, Vision and Eye Care at the University of South-Eastern Norway, Kongsberg, Norway and was approved by the Regional Committee for Medical Research Ethics for the Southern Norway Regional Health Authority. The participants were recruited from the National Centre for Optics, Vision and Eye Care and the Quality registry of inherited retinal diseases (IRD) at the Oslo University Hospital, Norway (14). Patients with genetically confirmed and/or clinically diagnosed adult-onset STGD1 (defined as age of onset ≥ 20 years) and logMAR visual acuity 0.4 or better when registered in the IRD quality registry, and healthy adults with normal vision and eye health were asked to participate. Written informed consent was obtained from all participants after explanation of the purpose and nature of the study. The Declaration of Helsinki was adhered to throughout the study.

Visual acuity (VA; in logarithm of the minimum angle of resolution units, logMAR) was measured with a digital high-contrast chart at 2.76 m (TestChart 2000; Thompson Software Solutions, London, United Kingdom). Ocular biometry was obtained using the IOL Master 700 (Carl Zeiss Meditec AG, Jena, Germany) to calculate the lateral scale of each retinal image.

### 2.2 Multimodal retinal imaging and analysis

The participants were imaged with color fundus photography (CFP; 45-degees; Canon CR-2 AF), fundus infrared (FIR), short wavelength-autofluorescence (FAF), spectral domain optical coherence tomography (SD-OCT), and confocal and non-confocal Adaptive Optics Scanning Light Ophthalmoscope (AOSLO).

#### 2.2.1 Optical coherence tomography

The Heidelberg Spectralis (Heidelberg Engineering GmbH, Germany) was used for SD-OCT, 30 degrees FIR and 30 and 55 degrees (short wavelength; 488 nm) FAF imaging (Spectralis Blue peak Module). SD-OCT volume scans (λ=880 nm; scan depth, 1.9 mm; 3.5 μm per pixel axial resolution in tissue; 14 μm per pixel lateral resolution in tissue) with enhanced depth imaging (EDI) mode enabled were acquired with the Spectralis OCT 2 Module. Ninety-seven B-scans (1536 A-scans per B-scan) were obtained across the central 30 x 20-degree area with 20 frames averaged during acquisition.

The retinal layers were segmented at the inner boundary of the inner limiting membrane (ILM), the posterior boundary of the outer plexiform layer (OPL), the center of the ellipsoid zone (EZ), and the posterior boundary of the RPE-Bruch’s Membrane (RPE-BrM) band, by using a semi-automatic active contour method, as described previously (15). Retinal thickness (RT) was calculated in microns as the distance between ILM and RPE-BrM and the thickness of the outer retinal layers (ORL) was calculated as the distance between OPL and RPE-BrM. Retinal layer-thicknesses were extracted from the segmented OCT images in areas of normal appearance on clinical imaging CFP, FIR and FAF and intact outer retinal structure on OCT, but with observed changes in the photoreceptor mosaic. The thickness values were also extracted over a 10-pixel region, 2.5-, 3.0- and 3.5 mm nasal to the foveal center, approaching the optic disc.

#### 2.2.2 Adaptive optics retinal imaging

Confocal reflectance (1), non-confocal split-detection (16) and dark-field (17) AOSLO images were acquired simultaneously through three imaging channels with the Kongsberg AOSLO (18), a custom-built instrument with 790 nm imaging beam and 850 nm wavefront sensing beam.

The participant’s pupil was dilated with tropicamide 0.5%, or cyclopentolate 1% in participants younger than 30 years, prior to imaging. Each participant was stabilized using a dental impression on a bite bar and instructed to fixate on a target consisting of a red dot of light positioned on a calibrated paper grid viewed through a dichroic mirror. The operator shifted the fixation target by 1 degree prior to capturing video sequences of the retina. Retinal locations up to 8° eccentricity were imaged at 1°-intervals using a 1° and 1.75° field of view to ensure overlap between the images. Each video sequence comprised of 150 frames and at least three sequences were taken at each retinal location for each participant. During the test, the operator encouraged the participant toward steady fixation and regular blinking (to prevent tear film break-up). The operator regularly adjusted both the focus and contrast, while also ensuring that the deformable mirror was regularly flattened to maintain consistent and acceptable image quality. The participant’s refractive error was fully or partially corrected with a spherical soft contact lens, if required to improve the quality of the images.

Sinusoidal image distortions were corrected by imaging a regular grid (Ronchi ruling) of known size at a known distance and then resampling the retinal images over a grid of equally spaced pixels. The raw images were processed using a horizontal strip registration method (19). Approximately 50 frames with the highest normalized cross correlation to a manually selected reference frame (with minimal distortions) from each image sequence were registered to improve signal-to-noise-ratio. We used a 4-surface eye model (20) for estimation of retinal magnification factor (RMF). If a contact lens was used in front of the eye while capturing retinal images, the RMF was scaled by a lens correction factor accordingly to Huang et al. (21).

The processed images were montaged semi-automatically using custom software to create in-vivo maps of the retina. By using a bespoke AO image alignment application, montages of the photoreceptor mosaic were aligned with en-face FIR, FAF and OCT scans using retinal landmarks (i.e. blood vessels, optic disc) after scaling each modality for differences in lateral magnification (20). The anatomical foveal center was first manually identified and marked on the OCT scan, as previously described (22), and then marked automatically in corresponding locations on the other imaging modalities. This was used to determine the retinal eccentricity for each region of interest.

**Figure 1:**
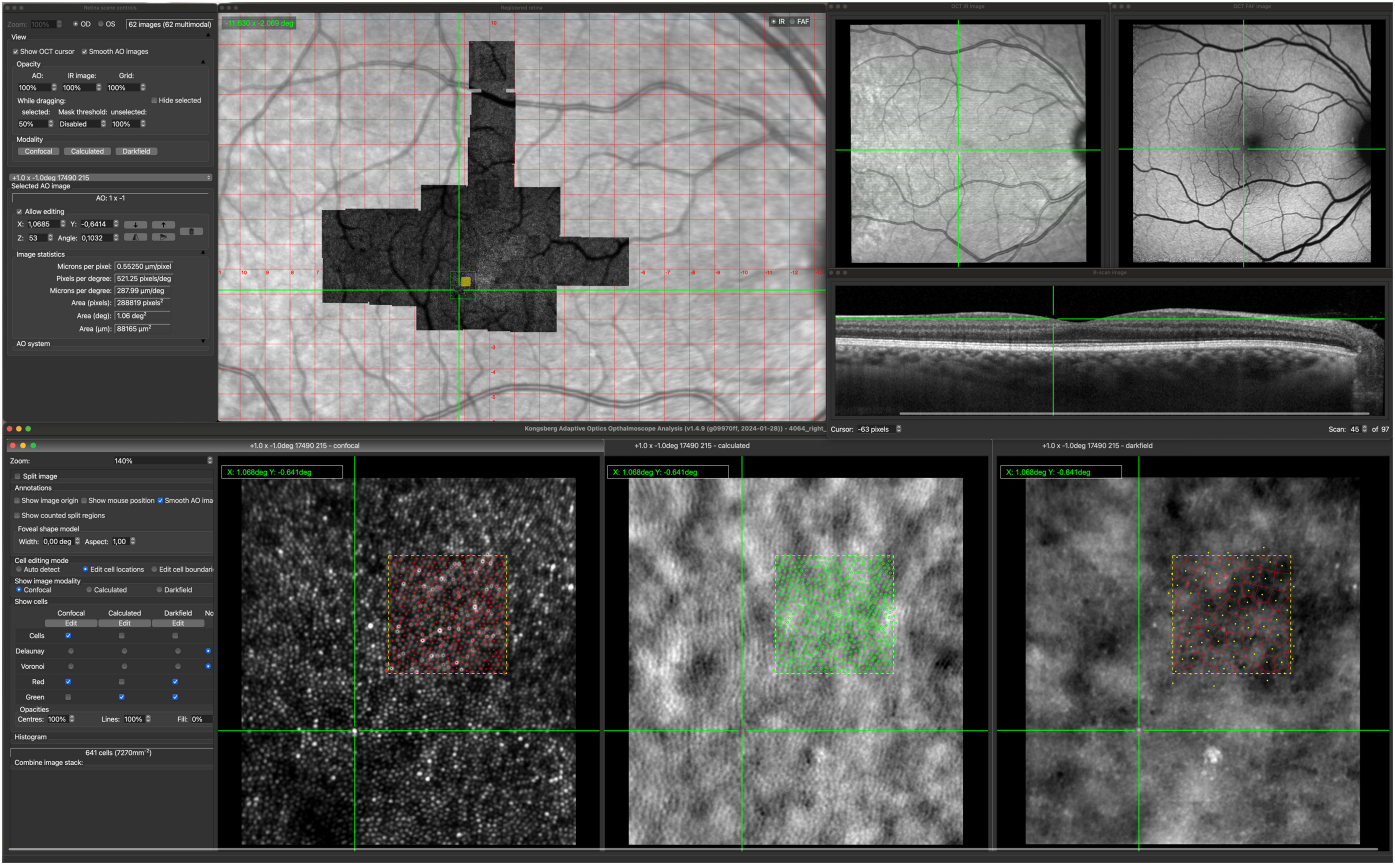
Kongsberg AOSLO Analysis user interface. The center of the green cross mark the corresponding locations on each of the image modalities. The green square marked on the AOSLO image montage show the location of the displayed confocal, non-confocal (calculated) and darkfield image. The filled yellow square on the AOSLO image montage marks the 100x100 µm region (yellow dotted squares) where reflective cones, cone inner segments and RPE cells are marked.

The software allowed via a single easy-to-use user interface: easy alignment of images; to explore changes in retinal structure across imaging modalities; to identify, delineate and correlate photoreceptors, RPE cells, and other retinal alterations in macular regions across imaging modalities (**Figure 1**). The software source code is open source and available from http://gitlab.com/cvri/kaoo_register.

Individual cones were identified and marked via a semi-automatic algorithm (23) on confocal AOSLO and manually on the corresponding split-detection AOSLO montages. RPE cells were identified and marked via a semi-automatic algorithm (24) on dark field images. Cell centers were used to generate Voronoi tessellations from which bounded cell densities and RPE cell areas were calculated. Cone and RPE density and spacing were extracted from regions of interest (50 x 50 µm size in the fovea, 100 x 100 µm in other macular areas) with acceptable quality for quantitative analysis.

#### 2.2.3 Data analysis

Descriptive methods, such as reports of mean, standard deviation (SD), median and range, were used to describe the demographics and clinical characteristics of the participants. Retinal features and structural changes in STGD1 were qualitatively described across imaging modalities. Eccentricity dependency in cone density was compared with RT and ORL, evaluated across patients and compared with data from healthy controls using Wilcoxon rank sum test or z-scores. The cone density values from the STGD1 patients were converted to z-scores (the number of standard deviations (SD) from the normal mean) based on the cone density at corresponding eccentricities in age-similar healthy eyes. Data from the best eye was used in the analysis. Statistical analyses were performed using R statistical software (25), version 4.2.1.

The intra- and interrater agreement of cone- and RPE-cell counting has been reported previously (2, 22). To show the intrarater reliability of the cone inner segment counting in the STGD1 patients in the current study, the inner segments were counted twice by the same rater (HRP) at least two months apart. Intra class correlation coefficient (ICC) estimate and its 95% confident interval were calculated using the R package “irr” (26) based on a two-way model with absolute agreement based on a single rater, and were found to be excellent (0.989 [0.955–0.998]).

## 3 Results

### 3.1 Clinical characteristics

Seven patients with clinically and genetically diagnosed adult-onset STGD1 (29–69 years, 3 males) and 18 healthy participants (23–74 years, 8 males) with normal vision and eye health were included in the study. The healthy control group had no known ocular pathology, no former intraocular or refractive surgery, and had corrected-to-normal visual acuity (-0.24 – 0.02 logMAR).

Clinical phenotype varied greatly across the patients with adult-onset STGD1. Visual acuity ranged from -0.02 to 0.98 logMAR. Demographics, clinical phenotype, and genotype for each STGD1 patient are shown in **Table 1**.

**Table 1:** Demographics, clinical phenotype, and genotype for each STGD1 patient.

Patient nr.	Age, years	Sex	Best eye	Axial length, mm	Visual acuity, logMAR	Age at symptom onset/ diagnosis, years	Clinical phenotype			Genotype, NM_00350.2 *ABCA4*
							Fundus photos (27)	FAF pattern (28)	Foveal sparing	
**1**	69	M	LE	24.46	-0.02	61/62	2	2	Yes	c.4685T>C, p.Ile1562Thr; c.4540-1000_4635-389delinsTGCCCG
**2**	29	M	RE	23.44	0.98	20/22	2	1	No	c.2588 G>C, p.Gly863Ala; c.3027del, p.Gln1010SerfsTer21
**3**	42	F	RE	25.42	0.70	35/35	3	3	No	c.71 G>A, p.Arg24His; c. 3682G>A, p.Glu1228Ter
**4**	52	F	LE	24.60	0.30	49/47	1	1	Yes	c.5882G>A, p.Gly1961Glu; c.5898 + 2T>C, splice
**5**	34	F	RE	23.41	0.84	24/29	1	1	No	c.3862 + 1G>A,splice; c. 5882G>A, p.Gly1961Glu
**6**	53	F	RE	23.06	0.16	40/48	2	2	Yes	c. 5461-10T>C, splice,T/C; c. 5603A>T, p.Asn1868IIe
**7**	35	M	RE	24.64	0.82	29/32	1	1	No	Not found*

*Clinical diagnosis Stargardt disease, mutation not found with whole exome sequencing with retina panel (7), sister has same phenotype. F, female; M, male; RE, right eye; LE, left eye.

### 3.2 Retinal structural changes associated with adult-onset STGD

Changes in retinal structure were visible in different image modalities depending on layer location and structural properties. **Supplementary Table 1** shows the qualitative description of the retinal features that we observed in adult-onset STGD1, across imaging modalities.

Cone and rod photoreceptors could be seen on confocal reflectance images but were easier to identify and quantify on non-confocal split-detection images. AOSLO image quality was not adequate to permit quantitative measures of the photoreceptor mosaic in one patient with large macular atrophy and unstable fixation, and in one patient with foveal sparing but poor tear film quality and subtle ocular media opacities. Ten of the healthy participants were included in the AOSLO analysis.

**Figure 2:**
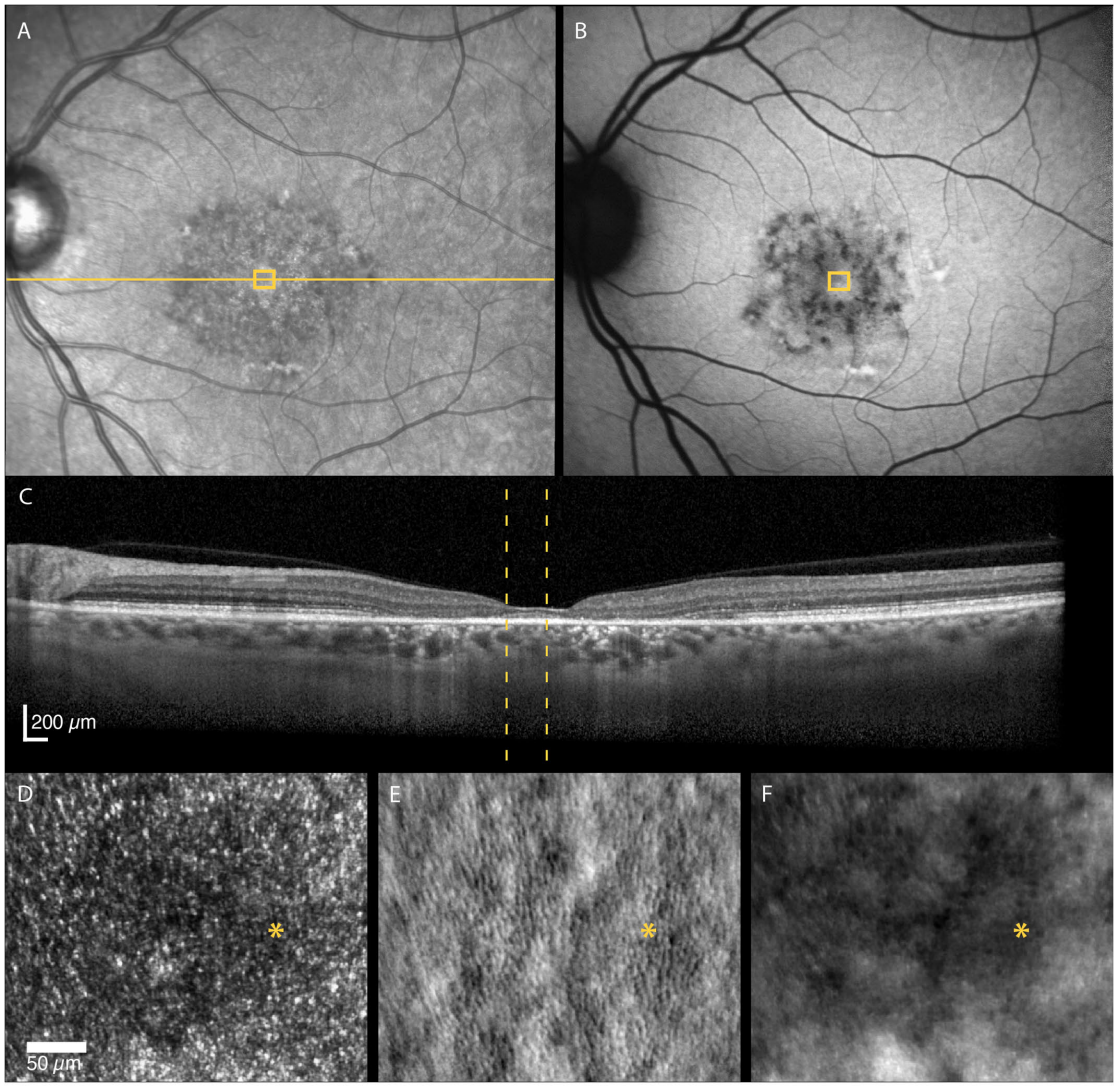
Multimodal imaging of a 52-year-old female with adult-onset STGD1 (Patient nr. 4). Intact outer retinal layers, referred to as foveal sparing, seen on OCT scan **(C)** of the foveal center corresponds to an area of residual cone inner-segment mosaic seen in the non-confocal split-detection image of the fovea **(E)**. Cone reflectance is irregular in the confocal image **(D)**. The RPE cell mosaic can be seen in the dark-field image **(F)**. The yellow solid line in **(A)** marks the OCT scan position **(C)**. The location marked by yellow boxes in **(A)** and **(B)** and yellow dotted lines in **(C)** corresponds to the AOSLO image location in **(D–F)**. The foveal center is marked with an asterisk.

The STGD1 patients had highly variable foveal structure, seen on FAF, OCT and non-confocal AOSLO, associated with equally variable visual acuity (**Table 1**). Foveal sparing was observed in three patients and was associated with intact outer retinal layers and residual cone inner-segment mosaic in or near the foveal center (**Figure 2**). Two of these patients had a late disease onset (≥ 45 years), typically associated with a foveal sparing phenotype (3). The foveal cone and RPE density could be reliably quantified in one of the patients with foveal sparing (Patient nr. 4) whose estimated foveal cone density (39 885 cones/mm^2^) was decreased compared to normal foveal cone density [104 985–163 797 cones/mm^2^, (2)]. In contrast, the estimated foveal RPE cell density (8298 cells/mm^2^) was within the normal range [6531–9285 cells/mm^2^, (2)]. Complete or incomplete foveal retinal and RPE atrophy with none or only a few remnant foveal cones were observed in four patients.

Using FAF and OCT, peripapillary sparing of the retina and RPE was seen in all patient eyes expect for one with STGD1 who had incomplete outer retinal atrophy (29) adjacent to the temporal edge of the optic disc.

#### 3.2.1 Non-waveguiding dark cones

**Figure 3:**
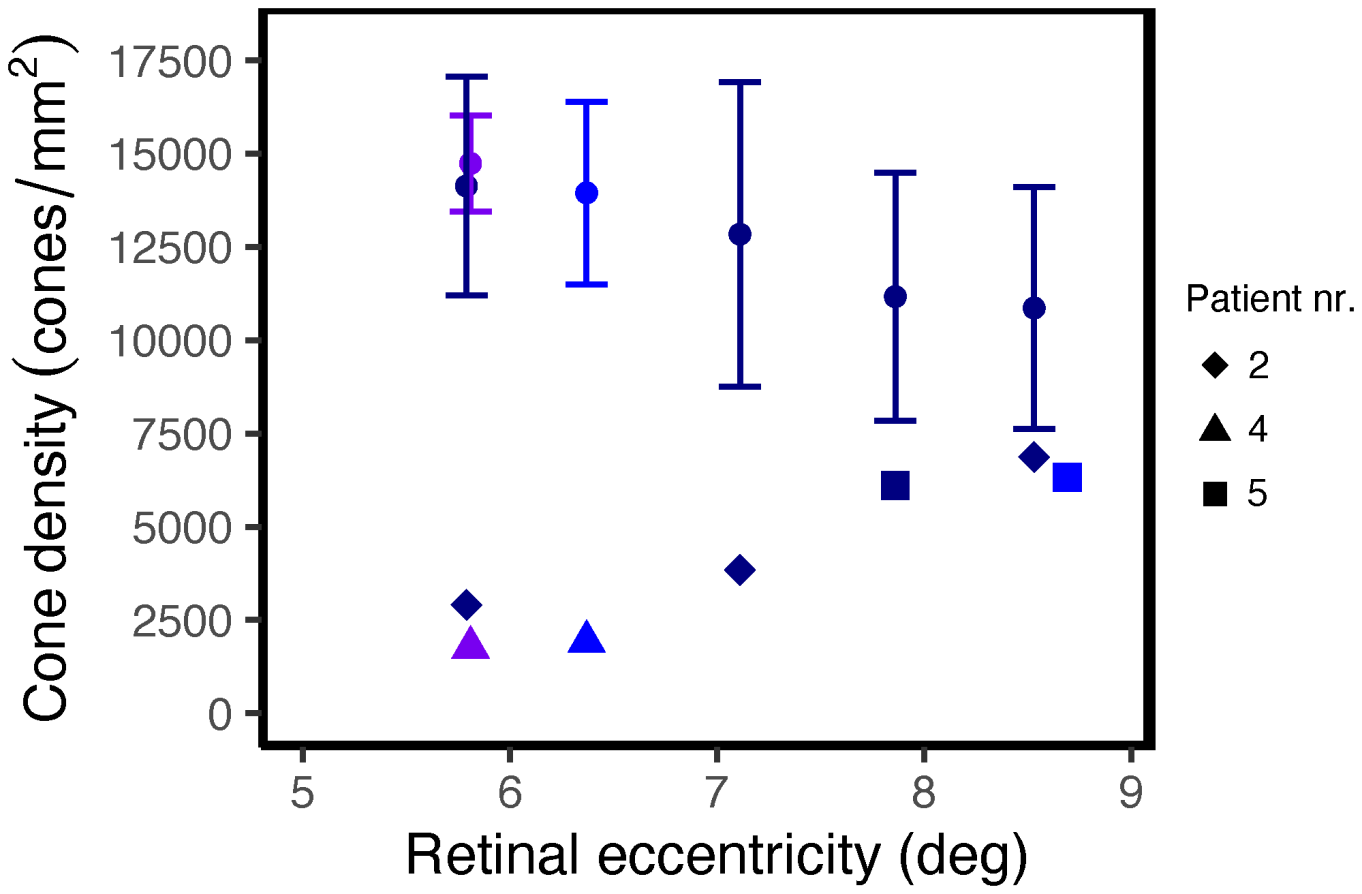
Cone density as a function of distance from the foveal center in regions with non-waveguiding cones in STGD1 patients. The round dots and error bars show the normal mean ± 2SD at the corresponding locations along the superior (dark blue), nasal (purple) or temporal (blue) meridian.

**Figure 4:**
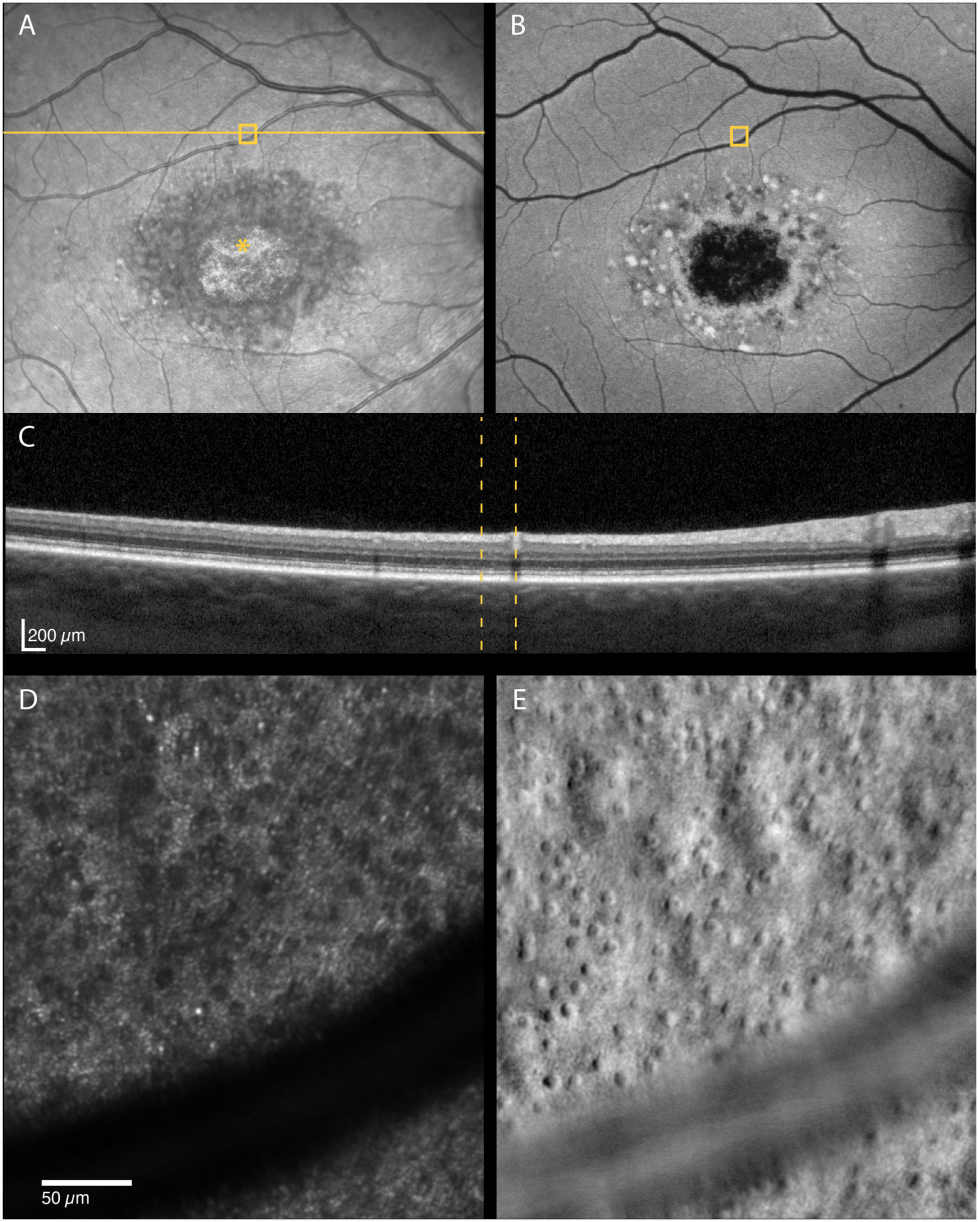
Multimodal imaging of a 29-year-old male with STGD1 (Patient nr. 2) showing regions of dark cones seen on confocal AOSLO **(D)** in areas with normal fundus infrared **(A)**, autofluorescence **(B)** and OCT **(C)** appearance. An intact cone inner segment mosaic can be seen in the corresponding non-confocal split-detection image **(E)**, although the cone density was lower than normal. The yellow solid line in **(A)** marks the OCT scan position **(C)**. The location marked by yellow boxes in **(A)** and **(B)** and yellow dotted lines in **(C)** corresponds to the AOSLO image location in **(D)** and **(E)**. The foveal center is marked with an asterisk.

**Figure 5:**
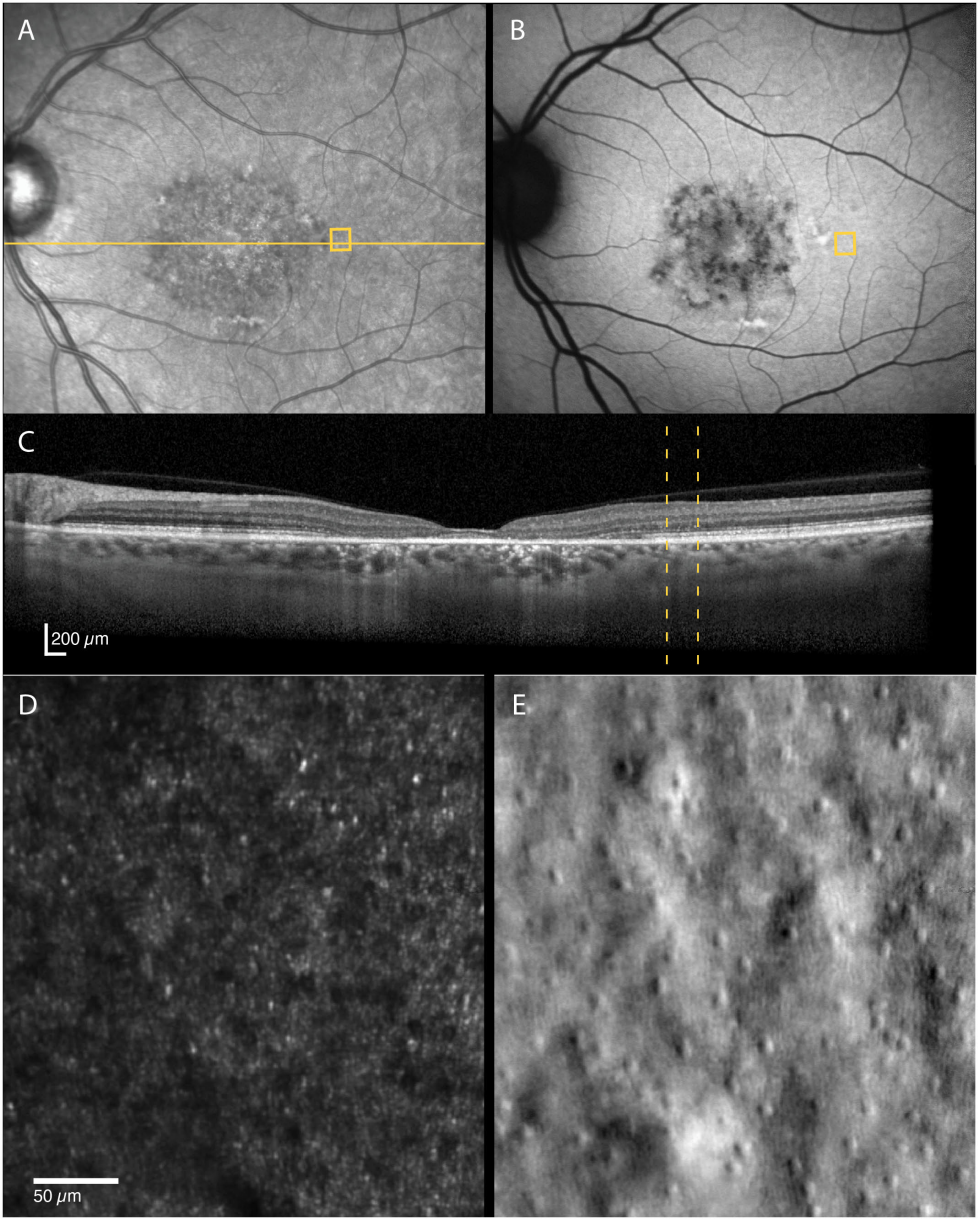
Multimodal imaging of a 52-year-old female with STGD1 (Patient nr. 4) showing regions of dark cones seen on confocal AOSLO **(D)** in areas with normal fundus infrared **(A)**, autofluorescence **(B)** and OCT **(C)** appearance. An intact cone inner segment mosaic can be seen in the corresponding non-confocal split-detection image **(E)**, although the cone density was lower than normal. The yellow solid line in **(A)** marks the OCT scan position **(C)**. The location marked by yellow boxes in **(A)** and **(B)** and yellow dotted lines in **(C)** corresponds to the AOSLO image location in **(D)** and **(E)**.

In retinal areas peripheral to the clinically apparent macular atrophic lesions, dark (hyporeflective) gaps in the photoreceptor mosaic were observed on confocal AOSLO in three patients. The dark gaps were surrounded by small hyperreflective spots probably corresponding to the rod mosaic. Most of these regions showed clinically normal CFP, FIR and FAF appearance and intact inner and outer retinal layers on OCT, including the EZ. We analyzed seven of the regions, of which three of these had reduced retinal thickness compared with the healthy controls (z-score < -2), while the outer retinal layers were thinner than normal in only two of these regions (z-scores -3.15 and -3.56). The non-confocal images showed an intact cone inner segment mosaic in all the corresponding regions, although cone density was reduced (1765–6867 cones/mm^2^, z-scores -2.45 to -20.21) compared with the healthy controls (8813–16 140 cones/mm^2^, **Figure 3**). The decrease in cone density was greatest in the regions closest to the fovea (**Figure 3**). Representative cases of areas with dark cones are illustrated in **Figures 4**, **5**. Dark cones were also observed in an area with a hazy, thickened ELM in one patient (Patient nr. 5).

#### 3.2.2 RPE-like structures visible on non-confocal split-detection AOSLO

**Figure 6:**
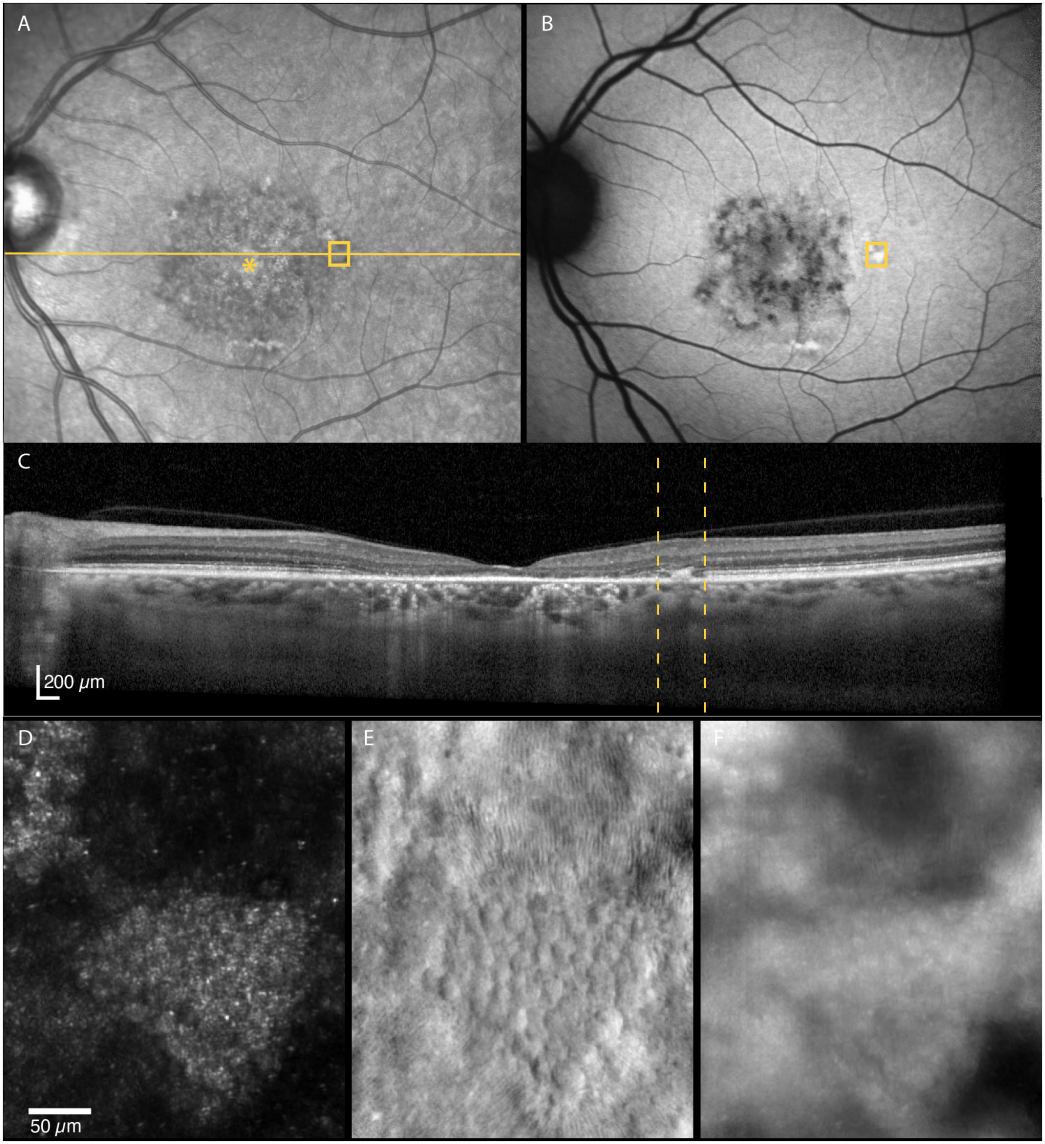
Multimodal imaging of an area near the transition zone between the atrophic and non-atrophic retina region (≈5 degrees temporal of the foveal center, **(A–E)**]. A small mosaic of polygonal structures, hypothesized to be RPE cells, is seen on the non-confocal split-detection image **(E)**. The corresponding location on the autofluorescence image shows a hyperreflective area **(B)**, whereas hyperreflectivity/thickening at the level of RPE-photoreceptor complex can be seen on OCT **(C)**. The RPE-like structures appear hyperreflective with darker edges on confocal reflectance **(D)** and dark field **(F)** images. The location imaged in **(D–F)** is indicated by yellow boxes in **(A)** and **(B)** and by yellow dotted lines in **(C)**.

A characteristic RPE cell mosaic was only occasionally visible on dark field images, most often close to the foveal center (**Figure 2**). In one patient (Patient nr. 4), non-confocal split-detection imaging showed polygonal structures that formed an orderly mosaic in an area near the transition zone between the atrophic and non-atrophic retina (≈5 degrees temporal, **Figure 6**). These structures were hyperreflective on dark-field imaging and FAF, and corresponded to hyperreflective thickening in the RPE-photoreceptor complex on OCT. Despite their abnormal reflectivity, the form of these structures was commensurate with the typical morphology of RPE cells (17, 30). The mean (SD) cell area (n=18 cells), 544.0 (71.0) µm^2^, was larger than the mean cell area reported for RPE cells at the similar retinal eccentricity, 398.6 (82.9) µm^2^, but was still within the normal range [(2), **Figure 3C**]. Similar structures could also be seen in three of the other STGD1 patients (patients 2, 6 and 7), but could not be quantified.

#### 3.2.3 Thickness of intact retinal layers on OCT

All the retinal layers were intact, and the retina appeared normal on CFP and FAF at 2.5- and 3.0-mm nasal in five of seven eyes with STGD, and at 3.5 mm nasal (adjacent to the temporal edge of the optic disc) in six of seven eyes. Retinal thickness was reduced compared with normal at 2.5 mm (median [range]: 276.4 [251.9–313.25] vs. 306.8 [285.5–332.4] µm, p = 0.024), and the outer retinal layers were thinner than normal at 2.5 mm (114.6 [106.7–143.6] vs. 131.0 [120.4–146.7] µm, p = 0.031) and 3.5 mm (107.3 [100.8–129.2] vs. 117.3 [110.2–127.0] µm, p = 0.036). Median RT and ORL thicknesses were like normal at the other analyzed locations (all p > 0.05).

## 4 Discussion

The implementation of multimodal in-vivo maps enabled direct comparison of macular degeneration in STGD1 across different imaging modalities while preserving the spatial relations between the structures. This allowed not only for identification of areas with non-waveguiding dark cones, but also quantification in relations to associated structures across different imaging modalities. We show that dark cones were in locations that have otherwise normal clinical appearance, including normal ORL thickness. Furthermore, the dark cones were aligned with an intact underlying cone inner segment mosaic, suggesting that this may be the first sign of retinal disease progression in adult-onset STGD1. Dark cones were also identified in an area with a thickened (and hazy looking) external limiting membrane. These two structural alterations — dark cones and thickened hazy external limiting membrane — have, on separate occasions, been described as early signs of retinal disease in childhood-onset STGD1 (11, 12). In the case of dark cones, these were only suggested to indicate loss-, or foreshortening of outer segments (12), as they did not include non-confocal imaging to confirm the presence of the inner segment mosaic. There is, however, one report showing that dark cones are aligned with underlying intact cone inner segments in a retinal area with abnormal thickness and non-intact interdigitation zone (IZ) in a young adult with STGD1 (31). Thus, our findings for adult-onset STGD1 corroborates that dark cones may be an early sign of retinal disease progression as reported in children and young adults with STGD1.

Intact cone inner segment mosaics underlying non-waveguiding cones have also been described in other inherited retinal diseases, such as achromatopsia [e.g. (16, 32, 33)], RDH5-associated fundus albipunctatus (34), oligocone trichromacy (35), retinitis pigmentosa and Usher syndrome (36). While the clinical implications of having dark cones need to be investigated further, the intact inner segments are suggested to be potential treatment targets in, for example, cone-directed gene or cell therapy (16).

Cone reflectivity can also be affected in other retinal diseases, such as AMD. Continuous mosaics of dark cones have not been reported in AMD, but several studies have described altered reflectivity at retinal locations overlaying drusen, subretinal drusenoid deposits and margins of geographic atrophy (18, 37–39). Outside the regions directly impacted by a lesion or atrophy, the distinctive waveguiding cone mosaic remains observable in non-neovascular AMD (40, 41). This is different from what is observed outside atrophic areas in the STGD1 patients in the present study. Therefore, it is reasonable to postulate that these disease specific cone phenotypes are a result of different disease mechanisms. The mechanisms that cause AMD are not confined to the photoreceptors (42), and damage to the RPE appear to be the first step of AMD (43). The observation of dark cones in locations with otherwise normal clinical appearance supports the growing evidence that, in STGD1, photoreceptor degeneration precedes clinically detectable RPE cell degeneration (12, 44, 45). The intact cone inner segment mosaic with reduced cone density (as assessed from non-confocal images), but normal RPE cell density at the same location as observed in the foveal center in one of the STGD1 participants (**Figure 2**), support this hypothesis.

The observed reduced cone density is consistent with other in-vivo imaging studies using AOSLO in STGD1, with reports of increased cone and rod spacing as well as enlarged cell size (12, 13, 31, 44). It is not known whether STGD1 patients have fewer and larger photoreceptors present at birth or whether photoreceptors degenerate early in disease before any other structural or functional changes are evident (12). AOSLO imaging of a densely packed foveal cone mosaic in young, asymptomatic STGD1 patients supports the latter, although cell density was not quantified (11).

Changes in thickness within the outer retinal layers has been suggested as an appropriate measure to detect early changes in retinal structure before they are clinically obvious (46). In STGD1, thinning of ORL within the macula (within ETDRS subfields and outside of EZ-loss) have been observed (46, 47). In this study, thinning of the intact retinal layers (RT and ORL) was found at the region closest to the area of EZ-loss (2.5 mm nasal), indicating loss of photoreceptor cells (46). Unfortunately, we cannot confirm this because AOSLO imaging was not performed at these eccentricities. Retinal layer thicknesses were normal at the more peripheral regions, except for the 3.5 mm region. Notably, thinning of the ORL was observed in this region temporal to the optic disc, which is inside the area reported as the spared region in STGD1 (48).

The multimodal in-vivo maps provided further insight into other structural changes that can be observed in STGD1. The polygonal structures quantified on non-confocal AOSLO images near the transition zone in one STGD1 patient are likely a cluster of RPE cells, although slightly enlarged compared with that seen in normal healthy individuals (2). Similar structures have previously been seen in STGD1 and were hypothesized to be hypertrophied RPE cells (31). It is uncommon to identify the RPE in this image modality, but it could be that its visibility is increased because of fewer overlaying photoreceptors. In addition, the corresponding hyperreflective thickening seen on OCT indicates anterior migration of RPE cells (**Figure 6C**). The dark-field images at the corresponding location show abnormal RPE mosaic reflectivity with hyperreflective centers and darker edges rather than the opposite which is what is expected from dark-field imaging of the normal RPE cell mosaic (2, 17, 49). The reason for this may be that the RPE cells contain accumulated material that has been associated with loss of transport function in ABCA4 retinal dystrophies (50).

This study has some limitations. First, the patient sample size was small. Second, some images were of insufficient quality for quantitative analysis of the photoreceptor and RPE mosaic. Thus, the data quantifying intact inner segments underlying dark cones are based on images from only seven locations in three STGD1 participants, and foveal cell density could be quantified in only one of the three patients with foveal sparing. Although better visual acuity correlated with the presence of foveal sparing, functional measurements in areas with dark cones (35) were not done, but this may shed light onto their clinical relevance in STGD1. A key strength of this study was the multimodal retinal imaging and use of custom software for alignment of the images and quantitative analysis of structural changes across six modalities via a single, easy-to-use user interface. Another strength of our study was the inclusion of age-matched control data taken with the same instruments and analyzed at corresponding retinal locations.

In conclusion, multimodal retinal imaging in-vivo maps is a valuable tool for identifying and quantifying structural alterations across different imaging modalities while preserving the spatial relations between the measures. Here, it was used to assess the presence of non-waveguiding dark cones in relation to intact inner segments at retinal locations that were quantified to be normal on OCT and with otherwise normal appearance on clinical imaging. This finding corroborates the hypothesis that dark cones are an early sign of photoreceptor degeneration and that in adult-onset STGD1 this may be the first sign of retinal disease progression. Longitudinal studies to follow the dark cones and their inner segments over time are warranted to confirm this and to see if it can be used to predict the rate of disease progression.

## Data availability statement

The original contributions presented in the study are publicly available in the USN Research Data Archive. This data can be found here: https://doi.org/10.23642/usn.25272964.v1.

## Ethics statement

The studies involving humans were approved by Regional Committee for Medical Research Ethics for the Southern Norway Regional Health Authority. The studies were conducted in accordance with the local legislation and institutional requirements. The participants provided their written informed consent to participate in this study.

## Author contributions

HP: Conceptualization, Formal analysis, Investigation, Methodology, Project administration, Validation, Visualization, Writing – original draft, Writing – review & editing, Software. SG: Data curation, Investigation, Methodology, Resources, Software, Writing – review & editing. LH: Investigation, Validation, Writing – review & editing, Project administration. JH: Resources, Writing – review & editing. RB: Resources, Writing – review & editing. RCB: Conceptualization, Funding acquisition, Methodology, Project administration, Resources, Supervision, Validation, Writing – review & editing, Software, Writing – original draft.
