# Supplementary material for: Multimodal in-vivo maps as a tool to characterize retinal structural biomarkers for progression in adult-onset Stargardt disease
Source: Front Ophthalmol (Lausanne). 2024 Apr 23;4:1384473. doi: 10.3389/fopht.2024.1384473 (PMC11182093; doi:10.3389/fopht.2024.1384473)
Supplement: Supplementary file 1 [file Table_1.docx]

Supplementary Material

**Supplementary table 1.** Qualitative description of different retinal features observed in adult-onset STGD1, across imaging modalities.

| **Feature** | **Color fundus photo** | **FIR image** | **FAF image** | **SD-OCT** | **AOSLO confocal image** | **AOSLO non-confocal split- detection image** | **AOSLO dark field image** |
| --- | --- | --- | --- | --- | --- | --- | --- |
| Flecks | Bright yellow-white appearance | Typically, hyperreflective structures, but can sometimes be seen with hypo-reflective borders. Resorbed flecks are typically hypo-reflective. | Hyperfluorescent, sometimes with hypo-fluorescent borders. Resorbed flecks are typically hypo-fluorescent | Hyperreflective deposits in the outer retina above the RPE | Highly reflective structures of different sizes and shapes | Bright-dark opposed edges that delineate round, oval, or amorphous structures on a grey background | Highly reflective structures of different sizes and shapes |
| Outer retinal atrophy | Not always visible, may be associated with a slightly darker orange color than surrounding fundus, sometimes a mottled appearance | Hypo-reflective area | Increased FAF, sometimes patches of diffusely decreased FAF giving a mottled appearance | ELM-, EZ- and IZ-reflective bands are disrupted or not visible | Undersaturated, no or few reflections. | No photoreceptor inner segments visible or very few inner segments observed occasionally |  |
| Outer retinal and RPE atrophy | Bright yellow/white area may look like «beaten bronze», choroidal vessels may be visible. Sometimes associated with pigment clumping. | Hyperreflective area, choroidal vessels may be visible. | Dark area with loss of FAF | RPE and ORL (ELM, EZ, IZ) is disrupted or missing, increased transmission of the SD-OCT signal into the choroid. | Bright, reflections in a random pattern, oversaturated. | No retinal cells visible. | Bright and dark reflections from the underlying choroid |
| Dark cones | Normal appearance, or some areas with flecks | Normal appearance, or hypo-reflective areas | Normal appearance or slightly increased FAF | Intact outer and inner retinal layers or visible thickening/hazy ILM | Dark (hypo-reflective) spaces in the photoreceptor mosaic, often surrounded by small reflective spots likely corresponding to the rod mosaic. | Visible cone inner segments that often are enlarged. Decreased cone density. | Hypo-reflective spots corresponding to the mosaic of the dark cones are sometimes visible. |
| Other features identified in any of the image modalities |  |  |  | Absence of the choroid in macular areas of with RPE and outer retinal atrophy | Hyperreflective polygonal RPE-like structures, with dark edges | Polygonal RPE-like structures just outside the transition zone | Hyperreflective polygonal RPE-like structures |

RPE, retinal pigment epithelium; FIR, fundus infrared; FAF, fundus autofluorescence; SD-OCT, spectral-domain optical coherence tomography; AOSLO, adaptive optics scanning light ophthalmoscope; ILM, inner limiting membrane, ELM, external limiting membrane; EZ, ellipsoid zone; IZ, interdigitation zone; ORL, outer retinal layers
